# Motivations, Behaviors and Expectancies of Sexting: The Role of Defensive Strategies and Social Media Addiction in a Sample of Adolescents

**DOI:** 10.3390/ijerph20031805

**Published:** 2023-01-18

**Authors:** Alessandra Ragona, Martina Mesce, Silvia Cimino, Luca Cerniglia

**Affiliations:** 1 Department of Dynamic, Clinical and Health Psychology, University of Rome, Sapienza, 00186 Rome, Italy; 2 Faculty of Psychology, International Telematic University Uninettuno, 00186 Rome, Italy

**Keywords:** sexting, defensive strategy, expectancies, social media addiction

## Abstract

Adolescents and young adults engage in sexting behaviors. Research has mainly emphasized the relationship between motivations and sexting behaviors, with little attention paid to sexting expectations and the potential role of coping strategies. This study aims to explore the measure of emotional–behavioral functioning with the Youth/Adult Self Report (based on the subject’s age), the use of defensive strategies measured with the Response Evaluation Measure (REM-71), social media addiction with the Bergen Social Media Addiction Scale (BSMAS) and all dimensions of sexting: motivations, behavior and expectations measured with the Sexting Motivation Questionnaire (SMQ), Sexting Behavior Questionnaire (SBQ) and Sexpectancies Questionnaire (SQ), respectively. *N* = 209 adolescents and young adults were recruited from high schools and universities in Rome to complete the self-report questionnaires. Results show that males tend to have higher expectations of sexting than females. We also found that expectations play a role in determining sexting behaviors and motivations. Our hypotheses on social media addiction and sexting were confirmed, while the relationship between the defensive strategies and sexting was not significant as expected. Further studies on this topic are desirable in the future.

## 1. Introduction

### 1.1. Sexting: Motivations, Behaviors and Expectancies

The term sexting—a combination of “sex” and “texting”—refers to the sending and/or receiving via digital media of sexually explicit messages and partial or fully nude photos and/or videos with sexual connotations [1,2].

Based on this general definition, we can find different forms of sexting: experimental sexting [3] and risky/aggravated sexting [4,5]. Wolak and colleagues [3] distinguish between “experimental sexting” as a way of exploring sexuality and “aggravated sexting” which implies exploitation and harassment of the other.

The first mentioned form of sexting, the experimental one, is an intimate form of communication in which sexual material is exchanged with the partner within an intimate relationship to explore sexuality; the second type of sexting, the risky one, is a high-risk form of communication in which sexting occurs simultaneously with other risky behaviors such as the use of substances and alcohol and/or engaging in sexting with strangers, increasing the probability of online dissemination of sexual material [3]. Recent research has shown that the tendency to sext is greater in youth who use alcohol and drugs [6] and meet strangers online [7]. It is important to note that sometimes young people use sexting to avoid negative emotions when they do not have the resources for more effective emotion regulation [8,9]. Furthermore, emotion dysregulation, emotional problems and negative emotions such as anger, loneliness and attention seeking are proven to predict risky sexting in young people [10].

It has been observed that sexting behaviors increase progressively in adolescence, reaching a peak in young adulthood [8,11]. The prevalent form of sexting in young adults is experimental sexting (47% to 77%), followed by the risky type (43%) and then by the emotional one (30%) [11,12,13].

Sexting is a phenomenon that has been increasing in recent years among teenagers and young people around the world, as demonstrated in several studies [14,15,16].

The distinction between experimental and aggravated sexting is possible thanks to the deepening of the underlying motivations, referring to a growing literature [17,18,19]. The most common form of sexting is related to sexual and social aims to experiment, to share within a romantic relationship or to initiate a sexual activity [14,15,16,18,20]. The motivations that instead regard risky sexting regard earning money, pleasing friends or a partner, or cyberbullying. Other reasons for sexting concern the reinforcement of body image within the peer group or peers’ approval of one’s appearance [21,22,23,24]; it possible to believe that sexting can be used to help build body image [25,26]. From a developmental perspective, creating a stable body image is an important task during adolescence [27]. In the hyperconnected contemporary society, this process also takes place through social networks and electronic media, leading the teenager to confront many more people than in the physical world and this can undoubtedly affect identity redefinition [28]. It is therefore possible to assume that sexting plays a role in increasing self-esteem and self-confidence related to body image [25].

The main categories of sexting motivations can also be connected to Lichtenberg’s motivational systems [29]: in particular, sexual motivations related to improving a romantic relationship can be linked to the need for attachment and affiliation; the reasons related to the reinforcement of body image, that is, the confirmation by peers about one’s body adequacy, may be the need for affiliation, exploration and assertion; finally, the motivations behind aggravated sexting could be linked to the need to react adversely through antagonism. In conclusion, motivations for sexting could represent an expression in cyberspace of innate motivational systems [20].

Regarding the expectations of sexting, a brief introduction to the theory of expectations is needed. Historically, the literature states that behavior is influenced not only by the antecedents of the behavior but also by the consequences [30]; according to the theory of expectations formulated by Vroom [31], consequences influence our behaviors. Expectations are related to the value of the outcome, understood as a positive or negative emotional evaluation of the outcome [32], but also to the belief that if a person enacts a behavior, the outcome that is believed will occur. This belief can be referred to as “expectancy” [33].

In most studies in the literature, expectations have been studied about alcohol [34], substance use [35] and, to return to the topic of this paper, condom use [36], but it is possible to apply the same concept to expectations about sexting behavior [14]. People who have positive expectations about sexting believe that sexting makes them more tender, intimate, more likely to have sex and also more attractive to others [14], and to feel more aroused, related to and loved [36]. On the other hand, negative expectations refer to feelings of disgust, shame and inappropriateness [14].

### 1.2. Sexting and Social Media Addiction

The development of new technologies, such as messaging, chat rooms and social media, has made communication easier but, at the same time, has raised a whole range of new issues. Adolescence is indeed a delicate period in which individuals are particularly influenced by the judgment of others; social networks seem to represent a fundamental role in the psychological and social development of young people [37] both because they constitute a considerable part of their lives but also because, by allowing immediate confrontation with peers, they also contribute to the maturation of identity [38]. The main risk an adolescent may face is that of developing an addiction [39]. Excessive use of social media is also believed to negatively affect general well-being and quality of life, especially in young users [40].

Social media thus represent a new medium for expressing oneself, socializing and sharing photos and videos: according to some, sexting has become an integral part of social exchange, used by adolescents to flirt, form romantic relationships, joke with friends and have fun [41,42,43]. This poses a risk because these tendencies could accelerate sexual development: this phenomenon is considered unhealthy because adolescents are still developing cognitively, socially and emotionally and therefore lack the maturity to understand [44].

Moreover, although sexting can be considered sometimes a normative form of sexual communication, it has been linked to signs of Internet and social media addiction and online gambling [11,45,46,47]. In fact, since adolescence represents a period of vulnerability, research states that adolescents are likely to engage in risky behaviors to show off and secure their social status: this could foster problematic Internet use, facilitating social media addiction and a higher prevalence of sexting among peers [48,49].

### 1.3. Defensive Strategies of Response and Behavioral/Emotional Problems: Role in Sexting

Adolescence is a period of change when the individual is most vulnerable and may develop psychopathological, emotional and behavioral problems [50].

To respond to stressful situations and protect oneself from intrusive feelings and thoughts [51,52], the individual resorts to coping and defensive strategies. These are unconscious reactions, not planned responses, that form an interface between innate temperament traits and learned coping strategies [53,54]. Defensive strategies can be adaptive when they maintain emotional homeostasis or dysfunctional when they limit mental functioning and distort reality [55]. Based on clinicians’ evaluations, Vaillant [56] presented a model of ego defensive strategies according to which ones can be arranged on a continuum of ego maturity, from immature to mature: While mature defensive strategies synthesize and attenuate stress, maintaining ego and reality in the process, immature ones deny and distort conflict.

This differentiation does not change with age, but the use of immature strategies is likely to be greater at a young age [57,58]. Indeed, the transition from the use of immature to mature defensive strategies is believed to be part of normal personality development [56,59]. Therefore, the standards for adequate defensive strategies functioning in adolescence differ from those in adulthood.

Some authors have suggested that sexting is a contemporary risk behavior that probably correlates with other risk behaviors [60]. Among the general population of high school students, adolescents who sext are more likely to be sexually active [61]; in addition, it has been observed that these young people also have less understanding of their emotions and more difficulty regulating them [60].

Therefore, it would be interesting to investigate the defensive strategy used by adolescents and young adults. In particular, it would be useful to observe the defensive strategies employed by adolescents concerning the dysfunctional use of new technologies that have become established in recent years and are now an integral part of daily life. Furthermore, since sexting is a complex phenomenon, the literature should study whether the use of this type of behavior is associated with specific defensive strategies or behavioral/emotional problems.

### 1.4. Aims of the Current Study

This study fits into a context of literature in which there are many studies on sexting but few to date have investigated the possible relationship between sexting and defensive structure from a psychodynamic perspective. Moreover, to our knowledge, few researchers have studied how these aspects of sexting (expectancies, motivations, behavior) are linked. For this reason, our study has focused on studying the relationships between these dimensions and verifying if there was a relationship with the defensive structure (measured with REM-71) or psychopathology (measured with Youth/Adult Self Report). Moreover, since social media have become an indispensable tool in the lives of young people, we also wanted to test the influence that social media addiction (BSMAS) has on sexting if a higher level of social media addiction corresponding to greater sexting behavior.

## 2. Materials and Methods

### 2.1. Participants and Procedure

The sample of the present study consisted of *N* = 209 subjects with *N* = 132 females (63%) and *N* = 77 males (37%); the sample age ranged from 14 to 25 years (M =18.8; SD = 3.55). We decided to use this age range consistent with the definition of “new adolescence” formulated by Sawyer and colleagues [62]. All subjects filled out an online randomized questionnaire through Google Forms after reading and accepting informed consent details; in the case of minors, the parents were permitted to participate. No personal information was requested in the study, and anonymity and confidentiality were guaranteed.

### 2.2. Measures

#### 2.2.1. Socio-Demographic Variables

Age, sex and sexual orientation were asked of participants. Sex was coded as a dummy variable (female = 0; male = 1). Sexual orientation involved the following options: heterosexual, homosexual, bisexual, asexual or other.

#### 2.2.2. Sexting Behavior Questionnaire (SBQ)

To assess sexting behaviors, the Sexting Behavior Questionnaire [15] was used, which is a modified version of the Sexting Behavior Scale [63]. This questionnaire consists of 29 items and investigates sexting behaviors in three sub-dimensions: receiving, sending and posting sexts on social media specifying who is the subject of the sexts and whether sexts were sent or posted with their consent. In this tool, sexting behaviors are defined as “sending or receiving sexually suggestive or provocative messages/photos/videos via mobile phone and/or Facebook or another Internet social networking site” and participants were assigned a score to each item from 1 to 5 (1 = never; 2 = rarely or a few times; 3 = occasionally; 4 = often; 5 = frequently or daily). In the present study, this scale showed an excellent internal consistency, with a Cronbach α value of 0.87.

#### 2.2.3. Sexting Motivations Questionnaire (SMQ)

The Sexting Motivations Questionnaire [25] was used to measure the three motivations for sending sexts. It is a 13-item self-report measure on a 5-point Likert-type scale from 0 (never) to 4 (always). This instrument measures the frequency of three sexting motivations: “sexual purposes” refers to sending sexts for sexual aims and it consists of five items. A sample item is: “Sometimes I send sexts for flirting or hooking up”; “instrumental/aggravated purposes” refers to the use of sexting for secondary aims, not related to sexuality, such as sharing sexts in exchange for something, under pressure/coercion or with harmful intentions and it consists of five items. A sample item is: “Sometimes I send sexts to hurt or damage someone”). The last motivation is “body image reinforcement” which applies when sharing sexts for getting positive feedback about your own body and it consists of three items. A sample item is: “Sometimes I send sexts to test whether I am attractive enough”. This tool showed good psychometric properties: Cronbach’s α obtained was α = 0.86.

#### 2.2.4. Sexpectancies Questionnaire (SQ)

The questionnaire is an adapted form of a measure created by Dir and colleagues [14] to assess expectations about the possible consequences of sending and receiving sexts.

The authors, to develop this measure, referred to the idea that people’s beliefs about sexting outcomes would be influenced by the expected outcomes for both the self and for other people [64]. This refers to the two different forms of learning, one’s own experiences and the socially learned experiences of those around the subject. Moreover, the authors have differentiated expectations based on receiving or sending sexts, as these are unique behavioral outcomes [14]. The instrument is composed of 49 items regarding possible outcomes of sexting behaviors on a Likert scale from 1 (not true at all) to 4 (extremely true). Participants received the definition of sexting at the beginning of the survey. The questionnaire consists of four subscales: positive and negative expectancies regarding sending sexts and positive and negative expectancies regarding receiving sexts. The “sending positive” and the “sending negative” subscales have, respectively, 18 and 10 items investigating the positive and negative emotions associated with sending sexts. The sending positive scale also contains items describing physical arousal and the likelihood of having sex. The “receiving positive” and the “receiving negative” subscales have, respectively, 10 and 11 items and consider negative emotions such as feeling “guilty”, “dirty” and “embarrassed”. Both the whole questionnaire and its subscales showed excellent internal consistency, with Cronbach’s α of 0.88 for the total scale and a range of 0.90–0.94 for the subscales.

#### 2.2.5. Bergen Social Media Addiction Scale (BSMAS)

The Bergen Social Media Addiction Scale (BSMAS) [65] consists of six items based on the six core components (salience, mood, modification, tolerance, withdrawal conflict and relapse) proposed by Griffiths [66] to evaluate social media addiction. Specifically, the items evaluate the experience of using social media on a 5-point Likert scale ranging between 1 (very rarely) and 5 (very often). A higher level of BSMAS corresponds to an increased risk of developing social media addiction. The cutoff score proposed by Bànyai [67] is 19 out of 30, following a large sample of Hungarian adolescents. Good psychometric properties of the BSMAS have been supported by different language versions (English [65], Italian [68], Persian [69], Portuguese [70]). In this study, Cronbach’s α obtained was α = 0.82.

#### 2.2.6. Response Evaluation Measure-71 (REM-71)

REM-71 [57] is a self-report questionnaire with 71 items for the assessment of defensive strategies. The questionnaire considers 21 defenses (Acting out, Splitting, Displacement, Dissociation, Fantasy, Passive aggression, Projection, Repression, Omnipotence, Undoing, Conversion, Somatization, Withdrawal, Suppression, Denial, Humor, Intellectualization, Reaction Formation, Idealization, Altruism, Sublimation) assessed with three or four items on a 9-point Likert scale from “1 = strongly disagree” to “9 = strongly agree”; high scores indicate a massive use of that specific defensive strategy. This questionnaire has a bifactorial structure along a mature–immature or adaptive–maladaptive axis. The total score of each subscale is given by the average of the items that compose it. Subjects who have completed the Italian version of the instrument [71] showed good internal consistency and test–retest reliability (reliability coefficient 0.84). It showed good psychometric properties in the present study; Cronbach’s α obtained was α = 0.91 for the total scale while the subscales obtained values that were in the range of 0.56 to 0.83.

#### 2.2.7. Achenbach System of Empirically Based Assessment (ASEBA): Adult and Youth Self Report

The Achenbach System of Empirically Based Assessment (ASEBA) comprises a family of forms for rating behavioral/emotional/social problems and adaptive characteristics [72]. Among these, the ASEBA also includes parallel forms completed by the assessed persons, including the Youth Self-Report (YSR) [73] for 11–18 year olds and the Adult Self-Report (ASR) [74] for 18–59 year olds that assess functioning in everyday settings over periods of 2–6 months [72]. Both are scored on a 3-point Likert scale (0 = not true; 1 = somewhat or sometimes true; 2 = very or often true) and include many subscales; in this study, we decided to employ the subscales “internalizing problems” and “externalizing problems”. While YSR consists of 112 items, ASR is composed of 123 items; both are provided for many cultures and have shown good reliability and validity [72]. In this study, YSR and ASR, respectively, achieved excellent internal consistency, with Cronbach’s α of 0.92 (YSR) and 0.96 (ASR) for the total scale and a range of 0.86 to 0.92 for the subscales of both.

### 2.3. Data Analysis

Preliminary statistical analyses were carried out using descriptive statistics such as the reliability of the measures, mean scores and percentages.

First of all, we focused on the relationship between sexting motivations, behavior and expectancies. To determine which subscale should be included in the regression analysis, a correlation between every dimension of sexting and its subscales was conducted. Any significantly related sext expectancy subscale was included in the regression to determine if it predicts the subscales of the sexting motivation questionnaire (Sexual purposes, Body Image Reinforcement, Instrumental/Aggravated Reasons) and sexting behavior.

Secondly, we conducted linear regression between the Social Media Addiction and sexting aspects to test our hypothesis, consistent with the literature [11,45,46,47], that a higher level of addiction predicted a higher greater tendency to sext.

Finally, to complete the model and verify if the behavioral/emotional problems and the defensive strategy had a role in sexting, we conducted separate linear regressions between the internalizing/externalizing subscales of Youth/Adult Self Report, Factor 1 and Factor 2 of REM-71 and SBQ, SMQ, SQ. The analyses were conducted using SPSS software (Version 21.0, IBM, Armonk, NY, USA).

## 3. Results

### 3.1. Sexting Motivations, Behavior and Expectancy

As regards the descriptive analyses concerning sexting motivations and sexting behavior, males have a higher score in almost all sexting dimensions except sexual purposes in which females have a slightly higher score (Table 1). However, conducting several ANOVAs we found that the only significantly different dimension is that of sexting behaviors where males have a higher score (SBQ score: female M = 33.8; male M = 36.5, *p* < 0.05, ηp^2^ = 0.027).

In addition, ANOVAs were conducted between males and females on sexting expectations. The results show a significance for two subscales of the Sexpectancies Questionnaire: negative expectations about sending sexts (*p* < 0.001, ηp^2^ = 0.059) and positive expectations about receiving sexts (*p* < 0.05, ηp^2^ = 0.022).

The correlation analyses between the SMQ, SBQ and SQ have shown the results summarized in Table 2. Briefly, positive expectations of sending are strongly related to all dimensions of sexting, motivational and behavioral (respectively: *p* < 0.001 with SBQ, SMQ_SEX and SMQ_BODY; *p* < 0.01 with SMQ_INS); positive expectations about receiving are strongly correlated with almost all sexting dimensions (respectively: *p* < 0.001 with SBQ and SMQ_SEX; with *p* < 0.01 with SMQ_BODY) except the instrumental/aggravated (SMQ_INS) type. The correlation between negative expectations on sending sexts was negative and significantly strong with almost all dimensions of sexting (in particular: *p* < 0.001 with SMQ_BODY; *p* < 0.01 with SBQ and SMQ_SEX) except the instrumental/aggravated (SMQ_INS) type. Finally, negative expectations about receiving were negatively correlated with one subscale of sexting motivations, sexual purposes (*p* < 0.001) and sexting behavior (*p* < 0.05).

Consistent with the study of Currin [75], we conducted regressions between SMQ, SBQ as regressors and SQ as a predictor. The detailed results in Table 3 show that sexting behavior is predicted only by positive expectations of sending (*p* < 0.001, R^2^ = 0.308, β = 0.57) and not from other expectations, making it possible to assume that higher positive expectations on the outcome favor engagement in a sexting behavior. The same result was obtained for sexting motivation subscales: sexual purposes (*p* < 0.001, R^2^ = 0.34; β = 0.47) and instrumental/aggravated (*p* < 0.001, R^2^= 0.04, β = 0.21). The motivation to strengthen body image is predicted both by positive expectations and by negative expectations of receiving.

The check for multicollinearity in every regression in the present paper was conducted by examining the Variance Inflation Factor (VIF). Conventionally, as suggested by Cohen [76] and O’Brien [77], VIF values greater than 4–10 generally indicate severe multicollinearity. Our results are VIF < 3, so multicollinearity is not an issue with our data. This section may be divided into subheadings. It should provide a concise and precise description of the experimental results, their interpretation, as well as the experimental conclusions that can be drawn.

### 3.2. Social Media Addiction and Sexting

Regarding analysis on social media addiction (female: M = 14.0 and DS = 5.59; male: M = 12.8 and DS = 5.10), the ANOVA between males and females did not show any statistically significant difference between the two groups.

Correlations show that the BSMAS is positively correlated with all dimensions of sexting (from *p* < 0.01 to *p* < 0.001) except instrumental/aggravated sexting and is positively correlated with positive expectancies about sending and receiving sexts (*p* < 0.001) (Table 4). Regression analyses were also found to be significant with SMQ and SBQ (*p* < 0.001, R^2^ = 0.09, β = 0.30) as dependent variables, highlighting that social media addiction has a role in determining sexting behaviors, sexting motivation for sexual purposes (*p* = 0.004, R^2^ = 0.04, β = 0.21) and body image reinforcement (*p* = 0.001, R^2^ = 0.05, β = 0.22). However, the effect size R^2^ is small [76,78].

In summary, it is possible to say that a higher level of social media addiction can lead to more motivation to sext and engage more frequently in sexting behavior.

### 3.3. Internalizing/Externalizing Problems, Defensive Strategy and Sexting

As regards the descriptive analyses on Youth/Adult Self Report and REM-71, the only statistically significant differences between males and females are given below. It emerges that females have higher levels in internalizing scales on youth/adult self-report (*p* < 0.001, ηp^2^ = 0.057), while at REM-71 males have slightly higher scores than females in both factor 1 and factor 2 but with a small effect (factor 1: *p* < 0.036, ηp^2^ = 0.021; factor 2: *p* < 0.035).

Secondly, we wanted to test our hypothesis that immature defensive strategies correlated with a more problematic type of sexting, such as instrumental/aggravated or body image reinforcement. To do this, we considered the two factors of REM-71 in correlation to each dimension of sexting motivations and behaviors. Correlation analysis found no significant relationship between sexting and factors 1 and 2 of REM-71. For this reason, from an exploratory perspective, we decided to investigate the individual subscales of REM-71, maintaining a more conservative *p*-value (*p* < 0.01). The results (Table 5) show the following relationship: fantasy (factor 1) and removal (factor 1) are correlated to sexting behaviors (SBQ); undoing (factor 1) is correlated to body image reinforcement; altruism (factor 2) is strongly and negatively correlated to instrumental/aggravated reasons.

This last result is particularly interesting because it suggests the idea that those who have a more altruistic defensive behavior are less inclined to engage in instrumental/aggravated sexting.

Finally, about internalizing and externalizing behaviors, it seems that only the externalizing scale has a relationship with sexting in motivations, expectations and behaviors. In fact, conducting linear correlations, externalizing behaviors are related to positive expectations, both sending (*p* < 0.001) and receiving (*p* < 0.001), sexting behaviors (*p* < 0.001) and sexual purposes (*p* < 0.05).

## 4. Discussion

Sexting is a phenomenon referring to the sending/receiving of sexually explicit messages, photos and videos [1,2]; generally, this is done via mobile devices: in recent years, in fact, this phenomenon has spread among adolescents and young adults worldwide [14,15,16]. Sexting can have both an adaptive function to strengthen the romantic and sexual relationship (sexual purposes) and a less adaptive function as a confirmation of the adequacy of one’s body (body image reinforcement) or to obtain favors from others (instrumental/aggravated) [17]. Sometimes young people use sexting to avoid negative emotions when they do not have the resources for more effective emotion regulation [8,9]. Moreover, it has been proven by some studies that emotional dysregulation, emotional problems and negative emotions (anger, loneliness and attention seeking) can predict risky sexting in young people [10]. In this broad panorama of studies on sexting, there are, however, some less explored areas such as the relationship between sexting expectations and motivations [33] and the possible relationship that a mature–immature defensive structure can have with sexting. Moreover, since sexting is a behavior that takes place online, it crucial to consider the influence that Internet social media addiction might have on sexting behaviors and some studies have linked it to signs of Internet and social media addiction and online gambling [11,45,46,47]. The purpose of this work was to investigate emotional–behavioral functioning, the use of defensive strategies, social media addiction and their relationships with sexting dimensions (motivations, expectancies and behavior). Based on recent discoveries in the literature [75], we first wanted to investigate the relationship between sexting expectations, motivations and behaviors, expecting an influence of expectations on other dimensions. Comparing males and females in sexting size, a significant difference emerges in sexting behaviors where males have a higher score than females and in two subscales of the Sexpectancies Questionnaire, negative expectations about sending sexts and positive expectations about receiving sexts. These data could be of particular importance to understanding how the image of oneself and others could be influenced by sexting expectancies. In fact, negative expectations about sending sexts refers to beliefs that people who do this activity are dirty, foolish and vulnerable and in contrast positive expectations about receiving refers to beliefs that people who receive sexts are admired, sexy and confident. In this context, young people could be influenced by these beliefs in engaging in or not sexting behaviors and in developing a negative idea of who does sexting. Male adolescents could also be more prone to engage in risky sexting behavior to appear admired and confident. This result seems to be in line with the current literature and in particular with the preliminary study about sexting expectancies conducted by Dir and Cyders [14] that found a higher sexting activity and higher positive expectations in males. In addition, the regressions carried out subsequently showed that the positive expectations about sending are predictors of all the motivations for sexting (sexual purposes, body image reinforcement and instrumental/aggravated) and also sexting behavior, as initially hypothesized. These data are consistent and in line with some findings in the literature that males send and receive more sexual messages [15,23], while females perceive riskier consequences, probably have had negative experiences and are under greater social pressure [6,43].

However, in addition to these results, the motivation to strengthen body image is predicted by both positive expectations about sending and negative expectations of receiving sexts, an unexpected outcome. It could be hypothesized that because an individual’s self-image is negatively affected by receiving or having received sexual messages, the individual might be more inclined to enact reassuring and reinforcing behaviors of an unsound self-image.

Moreover, our results do not confirm the recent literature results [75] that negative sending and receiving sexting expectancies predicted instrumental/aggravated motivations to sext. This might be an interesting point to investigate further as it seems the construct of aggravated sexting is more complex to define and understand than the other two types.

As for our next hypothesis regarding the influence of social media addiction on sexting, it seems confirmed by regression analyses, consistent with the literature on Internet addiction and sexting [11,45,46,47]. The results show that social media addiction has a role in determining sexting behaviors, sexting motivation for sexual purposes and body image reinforcement but has no influence on instrumental/aggravated type.

Finally, we tested our hypothesis that there was a relationship between mature–immature defensive functioning, emotional–behavioral functioning and sexting. We assumed that immature defensive strategies would be more associated with a less adaptive type of sexting as the reinforcement of body image and instrumental/aggravated type. Correlation analysis showed non-significant results between mature–immature defensive functioning and sexting. However, the analysis of the individual subscale of REM-71 brought interesting results: the dimensions “fantasy” and “removal”, both immature defensive strategies, correlate with sexting behaviors; “undoing”, an immature defensive strategy, correlates with body image reinforcement; and the most interesting result is that “altruism”, a mature defensive strategy, correlates negatively with the instrumental/aggravated type of sexting. From this result it is possible to speculate that higher levels of altruism are protective towards the implementation of risky sexting, providing an interesting basis for future insights into the protective role of a specific defensive strategy.

Finally, regarding emotional–behavioral problems, we found that only externalizing behaviors correlated with positive expectations, both sending and receiving, with sexting behaviors and sexual purposes. This could be due to a different self-perception in sexting individuals: sexting individuals may report poorer self-perceived behavioral conduct. These adolescents may likely feel judged and consequently judge themselves more negatively than their peers [79]. The co-construction theory also indicates that because there is an association between offline sexual behavior and externalizing problems (e.g., substance use [80,81]), sexting may therefore be related to externalizing symptoms.

### Limitations and Future Perspectives

The results of this study should be considered considering several limitations. First, the sample was collected in Rome with a convenient sample and this limits the generalization of the results to different groups of adolescents and young adults. For this reason, future research should focus on expanding the reference sample. Another possible limitation is due to the use of self-report questionnaires which may have led to a bias of social desirability and consequently an alteration of the results. Moreover, since the relationship between sexting behaviors, motivations and expectations seems to be in a very complex relationship, further research would be desirable to deepen this relationship and particular attention should be paid to instrumental/aggravated types of sexting that results in more difficult-to-understand sorts than other types.

## 5. Conclusions

Recent contributions on sexting in adolescents and young adults have emphasized the prevalence of the phenomenon and the importance of a better understanding of it and related variables, as it is an extremely complex phenomenon. However, most studies focus on personality traits related to the phenomenon and psychopathological traits. To our knowledge, few studies [75] have yet explored the complex relationship between sexting motivations and expectations and no study has considered the role of defensive strategies in sexting. In this paper, we sought to study these variables and their associations and observed that males tend to have higher expectations than females and that, in general, expectations play a key role in both motivations and behaviors related to sexting. In addition, social dependence was found to be a determining factor and the relationship with sexting should be further studied. Finally, the influence of defensive strategies on sexting is only in its infancy. This article aims to emphasize the importance of implementing knowledge on this topic to make psychoeducational program planning more effective. Indeed, we believe that a deeper knowledge of sexting and the mechanisms involved is crucial not only from a theoretical but also from a practical point of view in organizing more complex prevention and intervention programs for younger people. In this sense, in a future study perspective, it might be useful to further discriminate risk and protective factors as suggested by several authors [82,83] and it would be desirable to investigate how to implement different helping strategies taking into account the needs and characteristics of adolescents and young adults from the new knowledge that emerged in the literature [84].

## Figures and Tables

**Table 1:** Descriptive analysis of mean (standard deviation) and *p*-value between males and females in sexting behavior, motivation and expectancies.

	Males *N* = 77	Females *N* = 132	*p*-Value
SBQ	36.53 (9.2)	33.80 (7.1)	0.017 *
SMQ_INS	5.38 (1.0)	5.27 (0.6)	0.327
SMQ_SEX	12.38 (5.0)	12.89 (5.7)	0.520
SMQ_BODY	6.70 (3.4)	5.98 (3.2)	0.130
SQ_PS	44.79 (12.4)	43.06 (11.8)	0.317
SQ_NS	21.04 (7.1)	17.58 (6.4)	<0.001 ***
SQ_PR	24.95 (7.8)	22.53 (7.8)	0.033 *
SQ_NR	20.43 (7.2)	19.76 (6.7)	0.502

* *p* < 0.05, *** *p* < 0.001; SBQ = sexting behavior questionnaire; SMQ_INS = instrumental/aggravated sexting; SMQ_SEX = sexual purposes; SMQ_BODY = body image reinforcement; SQ_PS = positive expectancies on sending; SQ_NS = negative expectancies on sending; SQ_PR = positive expectancies on receiving; SQ_NR = negative expectancies on receiving.

**Table 2:** Correlations between sexting behavior, sexting motivations and sexting expectancies.

	1	2	3	4	5	6	7	8
1. SBQ	-							
2. SMQ_INS	0.500 ***	-						
3. SMQ_SEX	0.574 ***	0.191 **	-					
4. SMQ_BODY	0.603 ***	0.370 ***	0.500 ***	-				
5. SQ_PS	0.554 ***	0.217 **	0.575 ***	0.325 ***	-			
6. SQ_NS	−0.196 **	0.033	−0.306 ***	−0.040	−0.399 ***	-		
7. SQ_PR	0.420 ***	0.126	0.462 ***	0.188 **	0.759 ***	−0.205 **	-	
8. SQ_NR	−0.176 **	0.059	−0.244 ***	0.068	−0.361 ***	0.693 ***	−0.203 **	-

** *p* < 0.01, *** *p* < 0.001.

**Table 3:** Linear regression between sexting expectancies, motivations and behavior.

	SBQ			SMQ_INS			SMQ_SEX			SMQ_BODY		
Predictor	β	t	*p*	β	t	*p*	β	t	*p*	β	t	*p*
SQ_PS	0.575	5.925	<0.001	0.391	3.488	<0.001	0.474	5.001	<0.001	0.531	4.955	<0.001
SQ_NS	0.020	0.241	0.810	0.079	0.829	0.408	−0.118	−1.460	0.146	−0.036	−0.397	0.692
SQ_PR	−0.010	−0.111	0.912	−0.131	−1.252	0.212	0.082	0.931	0.353	−0.171	−1.716	0.088
SQ_NR	0.016	0.200	0.842	0.118	1.263	0.208	0.026	0.338	0.736	0.250	2.784	0.006

**Table 4:** Correlations between social media addiction and sexting.

	SBQ	SMQ_INS	SMQ_SEX	SMQ_BODY	SQ_PS	SQ_NS	SQ_PR	SQ_NR
BSMAS	0.301 ***	0.064	0.201 **	0.226 **	0.310 ***	−0−099	0.273 ***	−0−095

** *p* < 0.01, *** *p* < 0.001; BSMAS = Bergen Social Media Addiction Scale.

**Table 5:** Significant correlations between REM-71 subscales and SBQ, SMQ.

	1	2	3	4	5	6	7	8
1. SBQ	-							
2. SMQ_INS	0.500 ***	-						
3. SMQ_SEX	0.574 ***	0.191 **	-					
4. SMQ_BODY	0.603 ***	0.370 ***	0.500 ***	-				
5. REM_FANT	0.183 **	0.067	0.093	0.154	-			
6. REM_RE	0.254 ***	0.012	0.067	0.007	0.332 ***	-		
7. REM_UN	0.152	0.086	−0.011	0.179 **	0.458 ***	0.360 ***	-	
8. REM_AL	−0.152	−0.278 ***	−0.075	−0.033	0.002	0.115	0.288 **	-

** *p* < 0.01, *** *p* < 0.001; SBQ = sexting behavior questionnaire; SMQ_INS = instrumental/aggravated sexting; SMQ_SEX = sexual purposes; SMQ_BODY = body image reinforcement; REM_FANT = fantasy; REM_RE = removal; REM_UN = undoing; REM_AL = altruism.

## Data Availability

The data presented in this study are available on request from the corresponding author.

## References

[1] Chalfen R. (2009). ‘It’s only a picture’: Sexting,‘smutty’snapshots and felony charges. Vis. Stud..

[2] Currin J.M., Ireland M.E., Cox K., Golden B.L. (2020). Sextually aroused: A mixed-methods analysis of how it feels for romantic and sexual partners to send and receive sext messages. Comput. Hum. Behav..

[3] Wolak J., Finkelhor D., Mitchell K.J. (2012). How often are teens arrested for sexting? Data from a national sample of police cases. Pediatrics.

[4] Morelli M., Chirumbolo A., Bianchi D., Baiocco R., Cattelino E., Laghi F., Sorokowski P., Misiak M., Dziekan M., Hudson H. (2020). The role of HEXACO personality traits in different kinds of sexting: A cross-cultural study in 10 countries. Comput. Hum. Behav..

[5] Morelli M., Urbini F., Bianchi D., Baiocco R., Cattelino E., Laghi F., Sorokowski P., Misiak M., Dziekan M., Hudson H. (2021). The relationship between dark triad personality traits and sexting behaviors among adolescents and young adults across 11 countries. Int. J. Environ. Res. Public Health.

[6] Mori C., Temple J.R., Browne D., Madigan S. (2019). Association of sexting with sexual behaviors and mental health among adolescents: A systematic review and meta-analysis. JAMA Pediatr..

[7] Crimmins D.M., Seigfried-Spellar K.C. (2014). Peer attachment, sexual experiences, and risky online behaviors as predictors of sexting behaviors among undergraduate students. Comput. Hum. Behav..

[8] Bianchi D., Morelli M., Baiocco R., Chirumbolo A. (2021). Individual differences and developmental trends in sexting motivations. Curr. Psychol..

[9] Yoder J., Hansen J., Precht M. (2018). Correlates and outcomes associated with sexting among justice involved youth: The role of developmental adversity, emotional disinhibitions, relationship context, and dating violence. Child. Youth Serv. Rev..

[10] Ševčíková A. (2016). Girls’ and boys’ experience with teen sexting in early and late adolescence. J. Adolesc..

[11] Mori C., Cooke J.E., Temple J.R., Ly A., Lu Y., Anderson N., Rash C., Madigan S. (2020). The prevalence of sexting behaviors among emerging adults: A meta-analysis. Arch. Sex. Behav..

[12] Brodie Z.P., Wilson C., Scott G.G. (2019). Sextual intercourse: Considering social–cognitive predictors and subsequent outcomes of sexting behavior in adulthood. Arch. Sex. Behav..

[13] Bianchi D., Baiocco R., Lonigro A., Pompili S., Zammuto M., Di Tata D., Morelli M., Chirumbolo A., Di Norcia A., Cannoni E. (2021). Love in quarantine: Sexting, stress, and coping during the COVID-19 lockdown. Sex. Res. Soc. Policy.

[14] Dir A.L., Cyders M.A., Coskunpinar A. (2013). From the bar to the bed via mobile phone: A first test of the role of problematic alcohol use, sexting, and impulsivity-related traits in sexual hookups. Comput. Hum. Behav..

[15] Morelli M., Bianchi D., Baiocco R., Pezzuti L., Chirumbolo A. (2016). Sexting, psychological distress and dating violence among adolescents and young adults. Psicothema.

[16] Samimi P., Alderson K.G. (2014). Sexting among undergraduate students. Comput. Hum. Behav..

[17] Reed L.A., Boyer M.P., Meskunas H., Tolman R.M., Ward L.M. (2020). How do adolescents experience sexting in dating relationships? Motivations to sext and responses to sexting requests from dating partners. Child. Youth Serv. Rev..

[18] Levine D. (2013). Sexting: A terrifying health risk… or the new normal for young adults?. J. Adolesc. Health.

[19] Strohmaier H., Murphy M., DeMatteo D. (2014). Youth sexting: Prevalence rates, driving motivations, and the deterrent effect of legal consequences. Sex. Res. Soc. Policy.

[20] Bianchi D., Morelli M., Baiocco R., Cattelino E., Chirumbolo A. (2021). Patterns of love and sexting in teen dating relationships: The moderating role of conflicts. New Dir. Child Adolesc. Dev..

[21] Barrense-Dias Y., Berchtold A., Surís J.-C., Akre C. (2017). Sexting and the Definition Issue. J. Adolesc. Health Off. Publ. Soc. Adolesc. Med..

[22] Siibak A. (2009). Constructing the self through the photo selection-visual impression management on social networking websites. Cyberpsychology J. Psychosoc. Res. Cyberspace.

[23] Vanden Abeele M., Campbell S.W., Eggermont S., Roe K. (2014). Sexting, mobile porn use, and peer group dynamics: Boys’ and girls’ self-perceived popularity, need for popularity, and perceived peer pressure. Media Psychol..

[24] Van Ouytsel J., Van Gool E., Walrave M., Ponnet K., Peeters E. (2017). Sexting: Adolescents’ perceptions of the applications used for, motives for, and consequences of sexting. J. Youth Stud..

[25] Bianchi D., Morelli M., Baiocco R., Chirumbolo A. (2016). Psychometric properties of the Sexting Motivations Questionnaire for adolescents and young adults. Rass. Di Psicol..

[26] Hasinoff A.A. (2015). Sexting Panic: Rethinking Criminalization, Privacy, and Consent.

[27] Blos P. (1962). On Adolescence: A Psychoanalytic Interpretation.

[28] Schmitt D.P., Realo A., Voracek M., Allik J. (2008). Why can’t a man be more like a woman? Sex differences in Big Five personality traits across 55 cultures. J. Personal. Soc. Psychol..

[29] Lichtenberg J.D. (2013). Psychoanalysis and Motivation.

[30] Skinner B.F. (2019). The Behavior of Organisms: An Experimental Analysis.

[31] Vroom V.H. (1964). Work and Motivation.

[32] Behling O., Starke F.A. (1973). The postulates of expectancy theory. Acad. Manag. J..

[33] Goldman M.S. (1999). Expectancy operation: Cognitive–neural models and architectures. How Expectancies Shape Experience.

[34] Leigh B.C. (1990). The relationship of sex-related alcohol expectancies to alcohol consumption and sexual behavior. Br. J. Addict..

[35] Buckner J.D., Schmidt N.B. (2008). Marijuana effect expectancies: Relations to social anxiety and marijuana use problems. Addict. Behav..

[36] Currin J.M., Croff J.M., Hubach R.D., Miller B.M. (2017). Using sex-related alcohol expectancies to predict condom use among a general sample of men and women in the United States. Sex. Cult..

[37] Ramos-Soler I., López-Sánchez C., Torrecillas-Lacave T. (2018). Online risk perception in young people and its effects on digital behaviour. Comunicar. Media Educ. Res. J..

[38] Ragelienė T. (2016). Links of adolescents identity development and relationship with peers: A systematic literature review. J. Can. Acad. Child Adolesc. Psychiatry.

[39] Al-Samarraie H., Bello K.A., Alzahrani A.I., Smith A.P., Emele C. (2021). Young users’ social media addiction: Causes, consequences and preventions. Inf. Technol. People.

[40] Alzougool B., Wishah R. (2019). Use and addiction of social networking applications by university students in Jordan. J. Technol. Behav. Sci..

[41] Albury K., Crawford K. (2012). Sexting, consent and young people’s ethics: Beyond Megan’s Story. Continuum.

[42] Albury K., Crawford K., Byron P., Mathews B. (2013). Young People and Sexting in Australia: Ethics, Representation and the Law.

[43] Ringrose J., Gill R., Livingstone S., Harvey L. (2012). A Qualitative Study of Children, Young People and ’Sexting’: A Report Prepared for the NSPCC.

[44] Gabriel F. (2014). Sexting, selfies and self-harm: Young people, social media and the performance of self-development. Media Int. Aust..

[45] Hernández M.P., Schoeps K., Maganto C., Montoya-Castilla I. (2021). The risk of sexual-erotic online behavior in adolescents—Which personality factors predict sexting and grooming victimization?. Comput. Hum. Behav..

[46] Tamarit A., Schoeps K., Peris-Hernández M., Montoya-Castilla I. (2021). The impact of adolescent internet addiction on sexual online victimization: The mediating effects of sexting and body self-esteem. Int. J. Environ. Res. Public Health.

[47] Navas-Parejo M.R. (2020). Sexting among university students: Links to internet addiction and psychological variables. J. Drug Alcohol Res..

[48] Sesar K., Dodaj A., Šimić N. (2019). Motivational determinants of sexting: Towards a model integrating the research. Psihol. Teme.

[49] Gámez-Guadix M., De Santisteban P. (2018). “Sex Pics?”: Longitudinal predictors of sexting among adolescents. J. Adolesc. Health.

[50] Dahl R.E. (2004). Adolescent brain development: A period of vulnerabilities and opportunities. Keynote address. Ann. N. Y. Acad. Sci..

[51] American Psychiatric Association (1994). Diagnostic and Statistical Manual of Mental Disorders.

[52] Araujo K., Ryst E., Steiner H. (1999). Adolescent defense style and life stressors. Child Psychiatry Hum. Dev..

[53] Vaillant G.E. (1992). Ego Mechanisms of Defense: A Guide for Clinicans and Researchers.

[54] Perry J.C., Cooper S.H. (1992). What do cross-sectional measures of defense mechanisms predict. Ego Mechanisms of Defense: A Guide for Clinicians and Researchers.

[55] Ruuttu T., Pelkonen M., Holi M., Karlsson L., Kiviruusu O., Heilä H., Tuisku V., Tuulio-Henriksson A., Marttunen M. (2006). Psychometric properties of the Defense Style Questionnaire (DSQ-40) in adolescents. J. Nerv. Ment. Dis..

[56] Vaillant G.E. (1971). Theoretical hierarchy of adaptive ego mechanisms: A 30-year follow-up of 30 men selected for psychological health. Arch. Gen. Psychiatry.

[57] Steiner H., Araujo K.B., Koopman C. (2001). The response evaluation measure (REM-71): A new instrument for the measurement of defenses in adults and adolescents. Am. J. Psychiatry.

[58] Erickson S.J., Feldstein S.W. (2007). Adolescent humor and its relationship to coping, defense strategies, psychological distress, and well-being. Child Psychiatry Hum. Dev..

[59] Cramer P. (1991). The defense mechanism manual. The Development of Defense Mechanisms.

[60] Friedman S.H., Sorrentino R.M., Friedman J.B. (2017). Sexting: What are the clinical and legal implications. Curr. Psychiatry.

[61] Temple J.R., Paul J.A., Van Den Berg P., Le V.D., McElhany A., Temple B.W. (2012). Teen sexting and its association with sexual behaviors. Arch. Pediatr. Adolesc. Med..

[62] Sawyer S.M., Azzopardi P.S., Wickremarathne D., Patton G.C. (2018). The age of adolescence. Lancet Child Adolesc. Health.

[63] Dir A.L. (2012). Understanding Sexting Behaviors, Sexting Expectancies, and the Role of Impulsivity in Sexting Behaviors.

[64] Cooper M.L., Shapiro C.M., Powers A.M. (1998). Motivations for sex and risky sexual behavior among adolescents and young adults: A functional perspective. J. Personal. Soc. Psychol..

[65] Andreassen C.S., Billieux J., Griffiths M.D., Kuss D.J., Demetrovics Z., Mazzoni E. (2016). The relationship between addictive use of social media and video games and symptoms of psychiatric disorders: A large-scale cross-sectional study. Psychol. Addict. Behav..

[66] Griffiths M. (2005). A ‘components’ model of addiction within a biopsychosocial framework. J. Subst. Use.

[67] Bányai F., Zsila Á., Király O., Maraz A., Elekes Z., Griffiths M.D., Andreassen C.S., Demetrovics Z. (2017). Problematic social media use: Results from a large-scale nationally representative adolescent sample. PLoS ONE.

[68] Monacis L., de Palo V., Griffiths M.D., Sinatra M. (2017). Social networking addiction, attachment style, and validation of the Italian version of the Bergen Social Media Addiction Scale. J. Behav. Addict..

[69] Lin C.Y., Broström A., Nilsen P., Griffiths M.D., Pakpour A.H. (2017). Psychometric validation of the Persian Bergen Social Media Addiction Scale using classic test theory and Rasch models. J. Behav. Addict..

[70] Pontes H.M., Andreassen C.S., Griffiths M.D. (2016). Portuguese validation of the Bergen Facebook Addiction Scale: An empirical study. Int. J. Ment. Health Addict..

[71] Prunas A., Madeddu F., Pozzoli S., Gatti C., Shaw R.J., Steiner H. (2009). The Italian version of the response evaluation measure–71. Compr. Psychiatry.

[72] Achenbach T.M., Kreutzer J., DeLuca J., Caplan B. (2018). Achenbach System of Empirically Based Assessment (ASEBA). Encyclopedia of Clinical Neuropsychology.

[73] Achenbach T.M. (2001). Manual for ASEBA School-Age Forms & Profiles.

[74] Achenbach T.M., Dumenci L., Rescorla L.A. (2003). Ratings of Relations between DSM-IV Diagnostic Categories and Items of the Adult Self-Report (ASR) and Adult Behavior Checklist (ABCL).

[75] Currin J.M. (2022). Linking Sexting Expectancies with Motivations to Sext. Eur. J. Investig. Health Psychol. Educ..

[76] Cohen P., Cohen P., West S.G., Aiken L.S. (1983). Applied Multiple Regression/Correlation Analysis for the Behavioral Sciences.

[77] O’brien R.M. (2007). A caution regarding rules of thumb for variance inflation factors. Qual. Quant..

[78] Parker R.I., Brossart D.F., Vannest K.J., Long J.R., De-Alba R.G., Baugh F.G., Sullivan J.R. (2005). Effect sizes in single case research: How large is large?. Sch. Psychol. Rev..

[79] Kurup A.R., George M.J., Burnell K., Underwood M.K. (2022). A Longitudinal Investigation of Observed Adolescent Text-Based Sexting and Adjustment. Res. Child Adolesc. Psychopathol..

[80] Boislard P.M., Poulin F. (2011). Individual, familial, friends-related and contextual predictors of early sexual intercourse. J. Adolesc..

[81] Scott-Sheldon L.A., Carey K.B., Cunningham K., Johnson B.T., Carey M.P., MASH Research Team (2016). Alcohol use predicts sexual decision-making: A systematic review and meta-analysis of the experimental literature. AIDS Behav..

[82] Grov C., Breslow A.S., Newcomb M.E., Rosenberger J.G., Bauermeister J.A. (2014). Gay and bisexual men’s use of the Internet: Research from the 1990s through 2013. J. Sex Res..

[83] Benotsch E.G., Snipes D.J., Martin A.M., Bull S.S. (2013). Sexting, substance use, and sexual risk behaviour in young adults. J. Adolesc. Health.

[84] Bobkowski P.S., Shafer A., Ortiz R.R. (2016). Sexual intensity of adolescents’ online self-presentations: Joint contribution of identity, media consumption, and extraversion. Comput. Hum. Behav..

